# Physiological and metabolomics analyses of young and old leaves from wild and cultivated soybean seedlings under low-nitrogen conditions

**DOI:** 10.1186/s12870-019-2005-6

**Published:** 2019-09-06

**Authors:** Yuan Liu, Mingxia Li, Jingshu Xu, Xueying Liu, Shiyao Wang, Lianxuan Shi

**Affiliations:** Institute of Grassland Science, Northeast Normal University, Key Laboratory of Vegetation Ecology, Ministry of Education, Changchun, 130024 People’s Republic of China

**Keywords:** Gas exchange, Ionomics, Low nitrogen, Metabolomics, Old leaves, Soybean, Young leaves

## Abstract

**Background:**

It is critical to study the low nitrogen tolerance in wild soybean with extensive genetic diversity for improving cultivated soybean nitrogen use efficiency. Focusing on plant young and old leaves could provide new insights to low nitrogen tolerance research. This study compared the low nitrogen group with the control group on physiological and metabolomics changes in young and old leaves, respectively, then analyzed and compared the differences of these changes between cultivated and wild soybean. This study aimed to provide a theoretical basis for the molecular mechanism of soybean low nitrogen stress tolerance.

**Results:**

Wild soybean was less affected by low-nitrogen stress than cultivated soybean as assessed by plant biomass paraments, total carbon content and total nitrogen content. Gas-exchange coefficients and chlorophylls contents maintained relatively stable in wild soybean young leaves, but opposite in cultivated soybean. Wild soybean young leaves also increased the transport of beneficial ions, such as B^3+^, Fe^3+^, Mn^2+^, H_2_PO_4_^−^ and C_2_O_4_^2−^. In wild soybean old leaves, the nitrogen metabolism pathway was significant enhanced, especially the aspartic acid and GABA metabolisms. While in cultivated soybean, the nitrogen metabolism decreased obviously in young leaves but had no significant change in old leaves. The phenylpropanoid metabolism pathway was also activated in wild soybean. Contrary to cultivated soybeans, wild soybean tricarboxylic acid cycle and carbon metabolism including polyols and organic acids consolidated in old leaves and maintained a relative normal state in young leaves. These strategies could improve the antioxidant and N-fixation capacity in wild soybean.

**Conclusion:**

The survival and growth of wild soybean under low nitrogen stress conditions relied on physiological adjustments and metabolic changes that occurred at the cellular level. Compared with cultivated soybean, wild soybean young leaves could maintain a relatively normal growth mainly owing to a significant enhancement of key amino acids and nonprotein nitrogen metabolism in old leaves, especially aspartic acid, proline metabolism which provided basis for nitrogen reutilization from old leaves to young leaves. Consolidating the tricarboxylic acid cycle, intensifying phenylpropanoid metabolism, and accumulating more polyols and organic acids also had positive effect on it.

**Electronic supplementary material:**

The online version of this article (10.1186/s12870-019-2005-6) contains supplementary material, which is available to authorized users.

## Background

Nitrogen (N) is an essential nutritional element and its availability is extremely correlated with crop growth, yields and stress-responses [1]. Excess N compounds released from agricultural systems threaten the quality of air, water, and soil, which is also currently costing the European Union between €70 billion and €320 billion per year [2]. Improving ﻿plant N use efficiency is a significant challenge. Wild soybean (W, *Glycine soja*) is an important germplasm resource for studying stress resistance [3]. Wild soybean survives in natural selection and has unique physiological mechanisms for adapting to abiotic stress compared with cultivated soybean (C, *Glycine max*) [4]. It is critical to study low nitrogen (LN) tolerance strategy in wild soybeans with extensive genetic diversity for improving cultivated soybean N use efficiency.

Redistributions of ions and organic matters among different organs is essential for plant activities [5]. Many aspects of cellular metabolism have a direct impact on N metabolism activity in tissues, which is established through the combination of many genes, chemical balance and multi-layer regulation [6]. Nowadays, more and more researchers pay attention to the nitrogenous compounds reutilization under the stress conditions, especially low nitrogen condition [7]. Growth and physiological parameters at the seedling stage can be measured to determine abiotic stress-tolerance levels [8]. Metabolomics lends insight into the deep relationship between metabolites and changes in plant physiology conditions by combining a range of different analytical technologies and calculation methods [9]. In recent years, ionomics and metabolomics analyses have been widely used to determine responses to various abiotic stresses, including salinity, drought and nutritional deficits [10–12].

Numerous nutrient- and metabolic-based disorders, assessed in plant leaves and root, have been recorded under abiotic stress conditions [13]. While, the most studies used multiple leaf pools, giving no consideration to different positions and ages of the leaves. During the leaf growth, old leaf represent the final stage of leaf development and is characterized by the transition from nutrient assimilation to nutrient remobilization [14]. Plant Phloem-mobile nutrients redistribution is highly important for the economical use of nutrients under the stress [15]. The amino acids exported from old leaves may be utilized for the synthesis of constituents (e.g. enzyme, regulatory, or mem-brane proteins, nucleotides, and chlorophyll) in developing young leaves [16]. In Arabidopsis, phloem loading of nitrate in the source leaf and nitrate transport out of old leaves and into young leaves were rely on nitrate transporter NRT1.7 [17]. Meanwhile, a new study identified a genetic mechanism by young and old leaves differentially control stress-response cross-talk, this mechanism balances stress-response trade-offs to maintain plant growth and reproduction during stress [18]. Studies about the changes of metabolic processes include photosynthesis, respiration, nitrogen metabolism, sugar metabolism and fatty acid synthesis in young and mature leaves indicated that young and old leaves play different roles in response to abiotic stress conditions [19–21]. Therefore, focusing on plant young and old leaves could provide new insights to low nitrogen tolerance research.

To reveal the tolerant strategies in wild soybean to LN stress, the two experimental materials, W and C were subjected to low nitrogen treatment, and then the physiological and metabolite changes were compared between low nitrogen group and control group (CK) in the two soybean genotypes young and old leaves, respectively. Subsequently, we analyzed and compared the differences of these changes between cultivated soybean and wild soybean. Aim to provide a theoretical basis for the molecular mechanism of W tolerance to LN stress and the complex metabolic regulatory network involved in plant LN tolerance.

## Results

### Growth and photosynthetic characteristics

The LN stress had various significant effects on the two soybean genotypes performance. The biomass results showed C shoot height, root length, fresh weight and dry weight had a more significant inhibition than W (Additional file 1). Compared with CK, C young leaves total carbon content (%) decreased 11% significantly and total nitrogen content (%) decreased 10% significantly under the LN condition (*P* < 0.05); Under the same condition, W young leaves total carbon content (%) and total nitrogen content (%) had no significant changes (*P* > 0.05); C old leaves total carbon content (%) and total nitrogen content (%) had no significant changes (P > 0.05); W old leaves total nitrogen content (%) decreased significantly (*P* < 0.05) and total carbon content (%) had no significant changes (P > 0.05) (Table 1).

**Table 1:** Carbon and nitrogen contents in young and old leaves of two soybean varieties under LN stress

		Total content (%)				Fold changes Log_2_^(LN/CK)^	
		C		W			
		CK	LN	CK	LN	C	W
YL	Nitrogen	5.45 ± 0.09	4.91 ± 0.08	5.62 ± 0.16	5.13 ± 0.25	−0.15^*^	− 0.13
	Carbon	42.99 ± 0.26	38.24 ± 0.81	40.61 ± 1.19	38.63 ± 1.79	−0.17^*^	− 0.07
	C/N	7.89 ± 0.14	7.80 ± 0.30	7.23 ± 0.03	7.54 ± 0.03	− 0.02	0.06
OL	Nitrogen	4.49 ± 0.11	4.26 ± 0.05	5.01 ± 0.05	3.94 ± 0.14	−0.08	− 0.35^*^
	Carbon	41.84 ± 0.45	42.45 ± 0.26	40.36 ± 0.06	37.80 ± 0.85	0.02	− 0.10
	C/N	9.36 ± 0.33	9.97 ± 0.17	8.06 ± 0.08	9.60 ± 0.14	0.09	0.25^*^

Values were presented as the mean ± standard error of four biological replicates. *C* Cultivated soybean, *W* Wild soybean, *CK* Control treatment, *LN* Low nitrogen stress; *YL* Young leaves, *OL* Old leaves, *C/N* Carbon/Nitrogen; * indicate significant (*P* < 0.05)

**Fig. 1:**
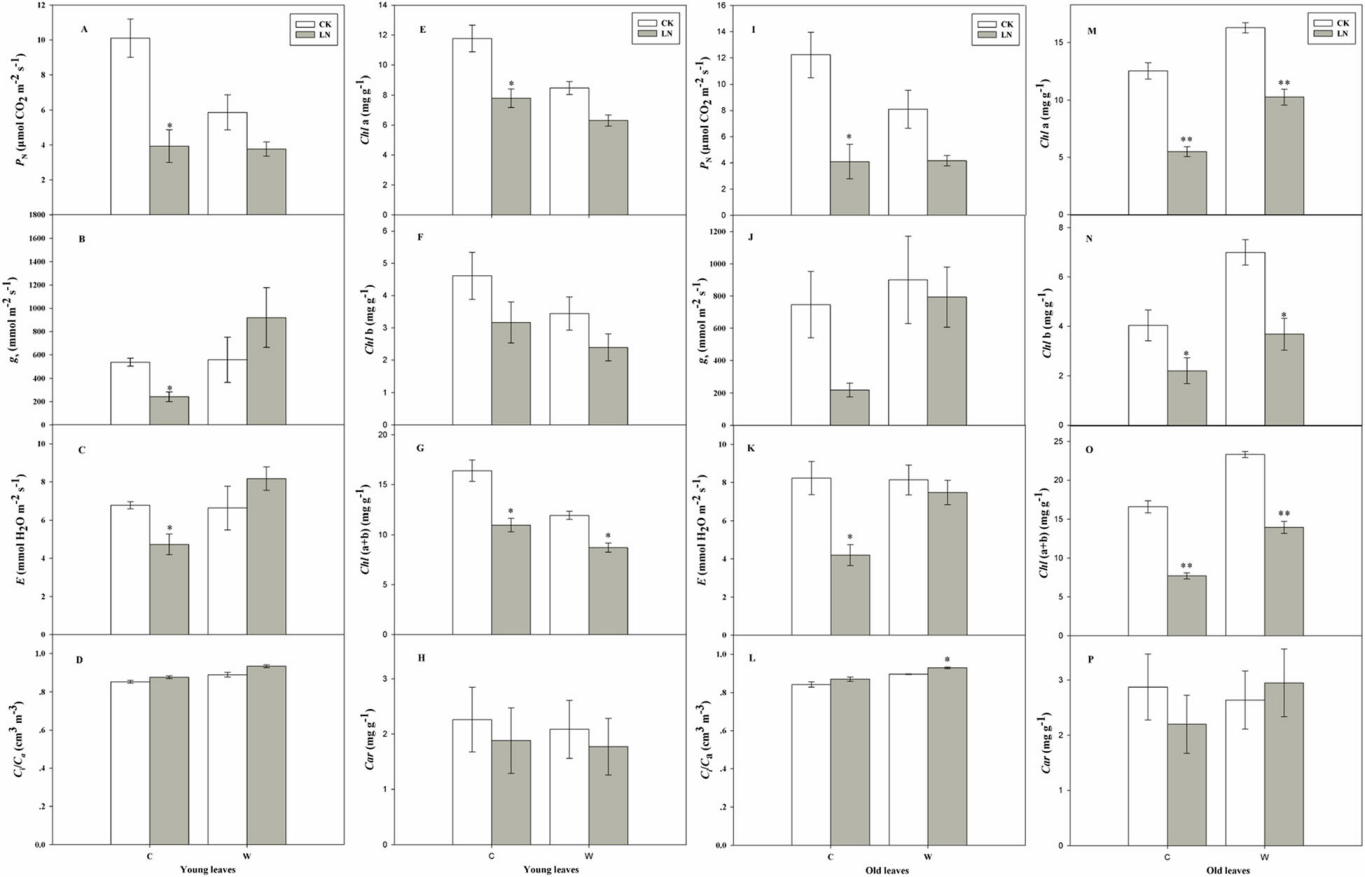
The changes in photosynthetic characteristics of the two soybean genotypes under CK and LN stress. (**a**) young leaves net photosynthetic rate (*P*_N_); (**b**) young leaves stomatal conductance (*g*_s_); (**c**) young leaves transpiration rate (*E*); (**d**) young leaves ratio of sub-stomatal to atmospheric CO2 concentrations (*C*_i_/C_a_); (**e**) young leaves chlorophyll a (*Chl* a); (**f**) young leaves chlorophyll b (*Chl* b); (**g**) young leaves chlorophyll a + chlorophyll b (*Chl* (a + b)); (**h**) young leaves carotenoid (*Car*); **(i**) old leaves net photosynthetic rate (*P*_N_); (**g**) old leaves stomatal conductance (*g*_s_); (**k**) old leaves transpiration rate (*E*); (**l**) old leaves ratio of sub-stomatal to atmospheric CO2 concentrations (*C*_i_/C_a_); (**m**) old leaves chlorophyll a (*Chl* a); (**n**) old leaves chlorophyll b (*Chl* b); (**o**) old leaves chlorophyll a + chlorophyll b (*Chl* (a + b)); (**p**) old leaves carotenoid (*Car*); C, cultivar soybean; W, wild soybean. CK, control treatment; LN, low-nitrogen stress; Error bars indicate the standard error (*n* = 4); * and ** indicate significant (*P* < 0.05) and highly significant (*P* < 0.01) differences, respectively

In C young leaves, compared with CK, the leaf net photosynthetic rate (*P*_N_), stomatal conductance (*g*_s_) and transportation rate (*E*) decreased significantly by 61, 55 and 30%, respectively (P < 0.05). In W young leaves, *P*_N_ decreased less than in C young leaves, meanwhile *g*_s_, *E* and ratio of sub-stomatal to atmospheric CO2 concentrations (*C*_*i*_ /*C*_*a*_) were increased, compared with CK. In the old leaves of both two genotypes, compared with CK, *P*_N_, *g*_s_ and *E* decreased under the LN stress, especially in C, that the *P*_N_, *g*_s_ and *E* values decreased by 66, 71 and 49%, respectively. Compared with CK, *C*_i_/*C*_a_ increased significantly under LN-stress conditions in W (Fig. 1). Compared with CK, *Chl* a, *Chl* b, *Chl* (a + b) and *Car* contents were all decreased in both C and W young leaves. The chlorophylls deceased more in C young leaves than in W young leaves. The similar trend also occurs in the old leaves of W and C. Compared with CK, *Chl* a, *Chl* b and *Chl* (a + b) contents decreased in W and C significantly (*P* < 0.05), especially in C old leaves. Car content was increased in W old leaves (Fig. 1).

### Ionomics responses

**Fig. 2:**
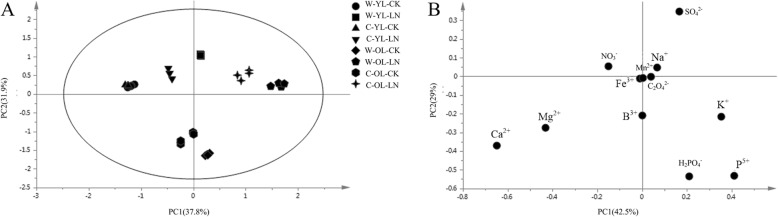
PCA of ionomic profiles and loading plots of ionomics in young and old leaves. (**a**) PCA of young and old leaves; (**b**) loading plot of young and old leaves; C, cultivar soybean; W, wild soybean; YL, young leaves; OL, old leaves; CK, control treatment; LN, low-nitrogen stress

According to the principal component analysis (PCA) of the ﻿ion contents in the two soybean genotypes young and old leaves, there were obvious differences in ionomics under LN-stress conditions (Fig. 2a). The young and old leaves were clearly separated by the first component (PC1), representing 37.8% of the total variation, and P^5+^, K^+^, Mg^2+^, Ca^2+^ and NO_3_^−^ were the major contributors. PC2 distinguished the control and LN stress groups, which represented 31.9% of the variation, and SO_4_^2−^, NO_3_^−^, H_2_PO_4_^−^ and P^5+^ were major contributors to PC2 (Fig. 2b; Additional file 2). In response to LN stress, compared with the CK, the contents of NO_3_^−^, Na^+^ and Mg^2+^ declined in the young leaves of the two soybean genotypes, especially in C, in which they significantly decreased by 54, 45 and 17%, respectively (*P* < 0.05). The contents of H_2_PO4^−^, C_2_O_4_^2−^, Mn^2+^, B^3+^, Fe^3+^, K^+^ and P^5+^ increased significantly in W young leaves (P < 0.05). In the old leaves, Ca^2+^, Mg^2+^ and NO_3_^−^ contents decreased in W and C. The contents of H_2_PO_4_^−^, SO_4_^2−^, Na^+^, B^3+^, Fe^3+^ and Zn^2+^ increased significantly in W old leaves (P < 0.05) (Table 2).

**Table 2:** Ion contents in young and old leaves of two soybean genotypes under LN stress

		concentration (mmol^.^ L^− 1^)				Fold changes Log_2_^(LN/CK)^	
		C		W			
		CK	LN	CK	LN	C	W
Young leaves	NO_3_^−^	2.78 ± 0.00	1.29 ± 0.00	2.26 ± 0.11	1.40 ± 0.01	−1.11**	−0.69**
	H_2_PO_4_^−^	16.23 ± 1.53	24.63 ± 0.30	18.57 ± 0.0.26	31.96 ± 0.97	0.60**	0.78**
	SO_4_^2−^	17.29 ± 0.42	18.14 ± 2.25	26.11 ± 0.20	23.59 ± 0.70	0.07	−0.15*
	C_2_O_4_^2−^	1.37 ± 0.02	1.47 ± 0.03	1.03 ± 0.06	1.72 ± 0.02	0.09*	0.74**
	Cl^−^	1.28 ± 0.01	4.48 ± 1.03	2.39 ± 0.11	4.47 ± 0.07	1.81	0.90
	Na^+^	2.79 ± 0.01	1.52 ± 0.06	2.15 ± 0.15	2.05 ± 0.05	−0.87**	− 0.07
	Mg^2+^	24.63 ± 1.29	20.39 ± 0.31	28.30 ± 0.79	26.48 ± 0.13	−0.27*	−0.10
	Mn^2+^	0.14 ± 0.01	0.12 ± 0.00	0.09 ± 0.00	0.13 ± 0.00	−0.15	0.59**
	B^3+^	0.33 ± 0.00	0.69 ± 0.01	0.35 ± 0.01	1.62 ± 0.00	1.05**	2.22**
	Fe^3+^	0.06 ± 0.00	0.08 ± 0.00	0.05 ± 0.00	0.12 ± 0.00	0.43**	1.17**
	Ca^2+^	11.66 ± 0.39	15.41 ± 0.39	14.61 ± 0.41	14.00 ± 0.05	0.40**	−0.06
	K^+^	144.34 ± 4.49	152.64 ± 4.49	191.66 ± 5.18	169.57 ± 0.30	0.08	−0.18*
	P^5+^	35.01 ± 1.78	39.01 ± 1.78	41.81 ± 0.68	57.06 ± 0.13	0.16	0.45**
	Zn^2+^	0.16 ± 0.01	0.21 ± 0.00	0.14 ± 0.01	0.24 ± 0.00	0.39	0.78
Old leaves	NO_3_^−^	4.94 ± 0.04	1.08 ± 0.04	3.96 ± 0.04	0.92 ± 0.04	−2.19**	−2.10**
	H_2_PO_4_^−^	19.97 ± 0.14	27.34 ± 0.42	20.58 ± 0.05	46.04 ± 0.66	0.45**	1.16**
	SO_4_^2−^	12.28 ± 0.15	28.04 ± 0.87	2.16 ± 0.12	2.79 ± 0.11	1.19**	0.37**
	C_2_O_4_^2−^	1.05 ± 0.04	1.14 ± 0.04	1.33 ± 0.00	1.34 ± 0.04	0.12	0.01
	Cl^−^	2.25 ± 0.11	8.46 ± 0.67	2.49 ± 0.18	7.77 ± 0.24	1.91	1.64
	Na^+^	1.03 ± 0.03	2.40 ± 0.51	1.01 ± 0.04	1.82 ± 0.06	1.22*	0.85**
	Mg^2+^	45.05 ± 0.75	38.95 ± 1.48	46.40 ± 0.01	33.28 ± 0.06	−0.21**	− 0.48**
	Mn^2+^	0.17 ± 0.01	0.19 ± 0.04	0.12 ± 0.02	0.16 ± 0.04	0.21	0.46
	B^3+^	0.86 ± 0.02	5.21 ± 0.12	1.09 ± 0.03	4.86 ± 0.05	2.60**	2.16**
	Fe^3+^	0.10 ± 0.03	0.09 ± 0.00	0.04 ± 0.00	0.09 ± 0.00	−0.19	0.98**
	Ca^2+^	59.14 ± 0.55	48.22 ± 0.70	48.96 ± 0.14	31.32 ± 0.07	−0.29**	− 0.64**
	K^+^	152.58 ± 2.48	139.95 ± 3.09	163.44 ± 0.07	172.59 ± 0.70	−0.12*	0.08**
	P^5+^	27.36 ± 0.05	47.08 ± 0.88	32.87 ± 0.12	70.81 ± 0.04	0.78**	1.11**
	Zn^2+^	0.08 ± 0.00	0.25 ± 0.01	0.09 ± 0.00	0.35 ± 0.00	1.64	1.96

Values were presented as the mean ± standard error of four biological replicates. *C* Cultivar soybean, *W* Wild soybean, *CK* Control treatment, *LN* low−N stress. * and ** indicate significant (*P* < 0.05) and highly significant (*P* < 0.01) differences, respectively

### Metabolomics analysis

**Fig. 3:**
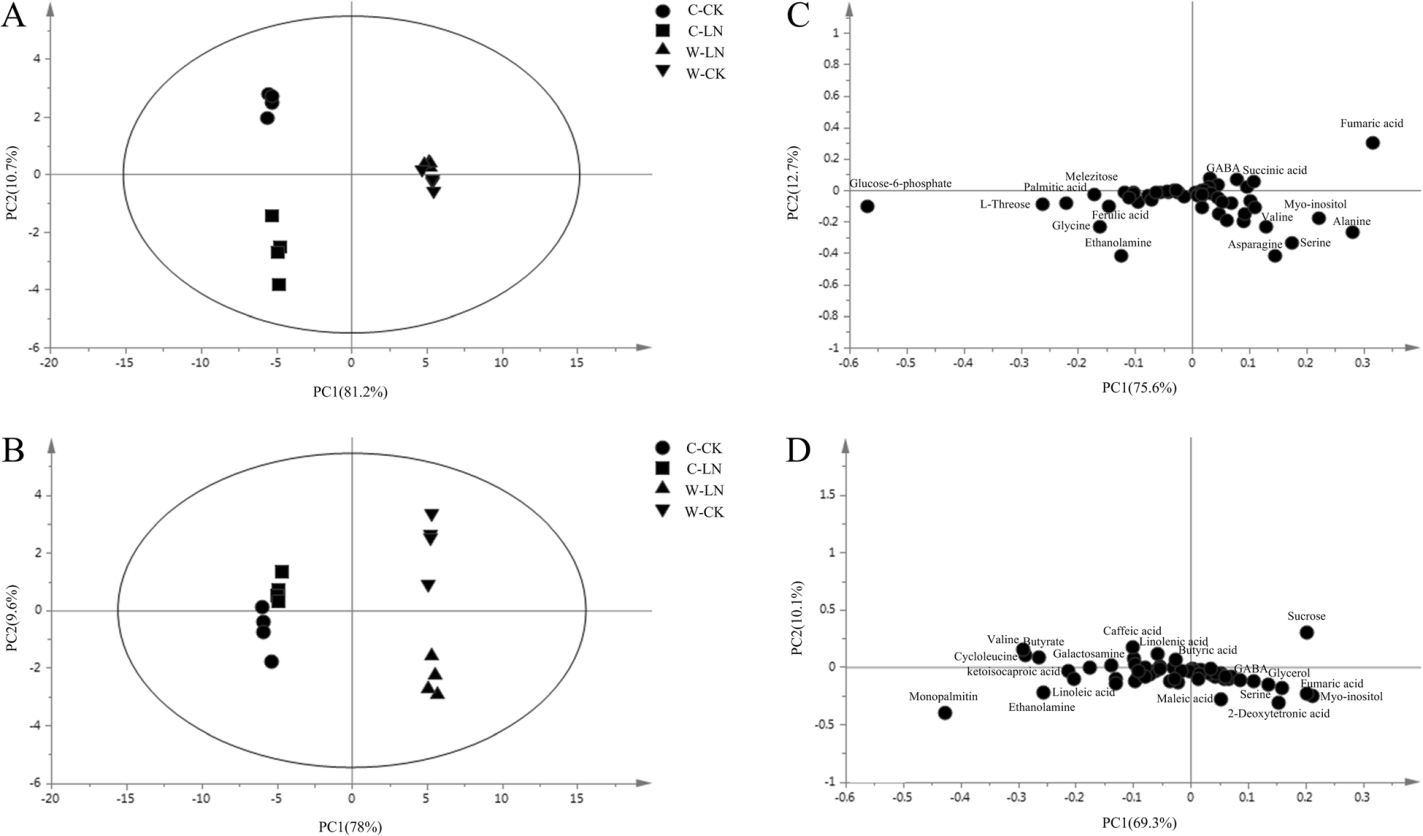
PCA of metabolic profiles and loading plots of metabolites in young and old leaves. (**a**) PCA of young leaves; (**b**) PCA of old leaves; (**c**) loading plot of young leaves; (**d**) loading plot of old leaves. C, cultivar soybean; W, wild soybean; CK, control treatment; LN, low-nitrogen stress

We conducted a PCA on the detected differential metabolites to identify the key factors affecting the metabolomics (Fig. 3; Additional file 3). PC1 explained 81.2% of the variance in young leaves and 78% of the variance in old leaves, which predominantly reflected the difference between W and C; PC2 distinguished the CK and LN-stress groups that explained 10.7% of the variance in young leaves and 9.6% of the variance in old leaves, indicating that LN stress had a substantive effect on the metabolites (Fig. 3a;b). The contribution of metabolites in young leaves to PC1 was dominated by fumaric acid, alanine, serine, myo-inositol and glucose-6-phosphate, while ethanolamine, asparagine, serine, valine and fumaric acid were major contributors to PC2 (Fig. 3c; Additional file 4). The contributions of metabolites in old leaves to PC1 came several metabolites, dominated by monopalmitin, fumaric acid myo-inositol, sucrose, valine and butyrate, while caffeic acid monopalmitin and valine were the dominate metabolites contributing to PC2 (Fig. 3d; Additional file 5).

**Fig. 4:**
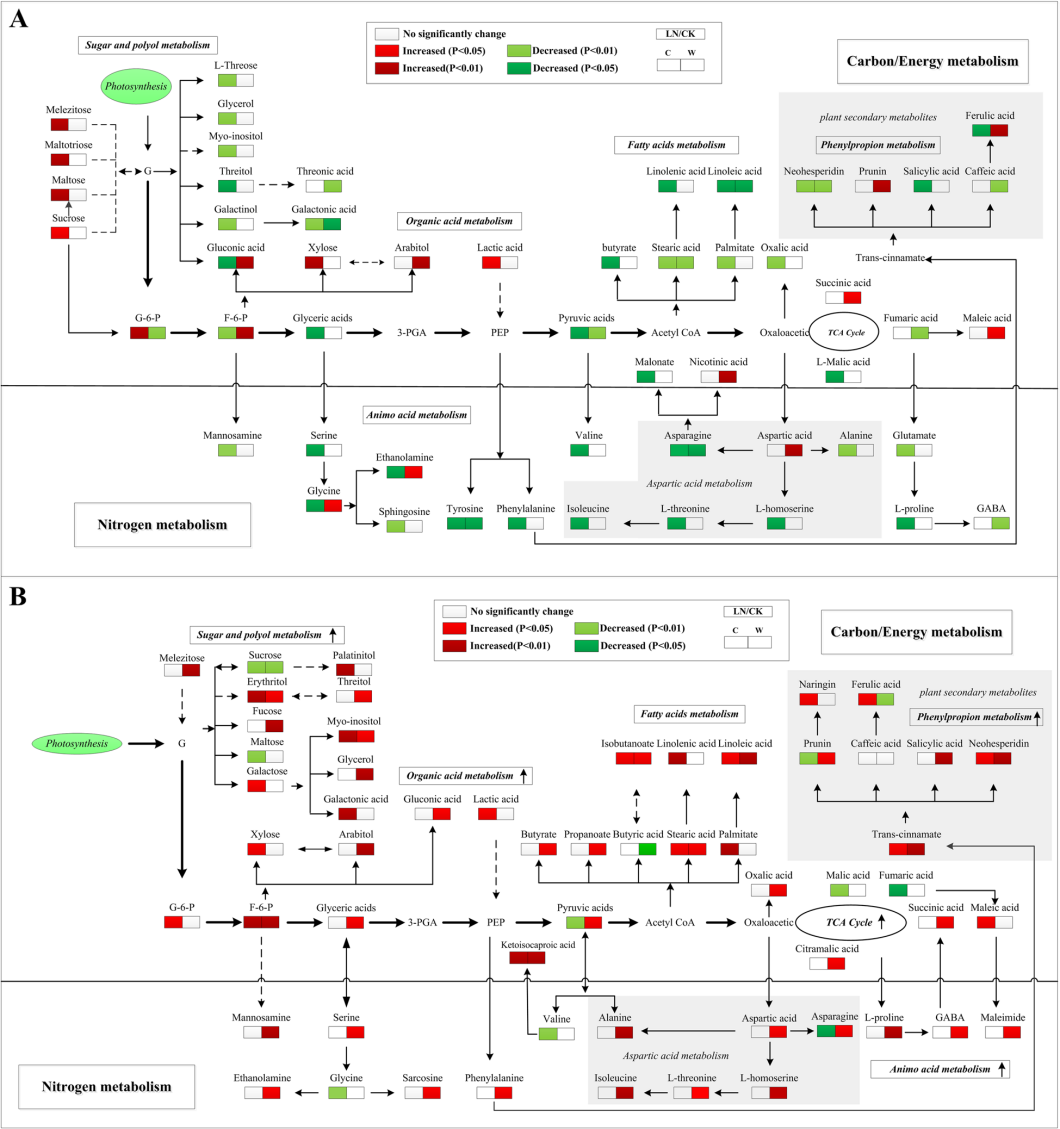
Changes in the metabolic pathways of young and old leaves in the two soybean genotypes. Suggested changes in the metabolic network in soybean seedlings under LN-stress conditions based on a partial least square-discriminant analysis (PLS-DA). (**a**) pathway of young leaves; (**b**) pathway of old leaves. C, cultivar soybean; W, wild soybean; CK, control treatment; LN, low-nitrogen stress

The levels of 54 differential metabolites of young leaves in W and C were independently calculated and compared (Table 3; Fig. 4a). In the young leaves, the content of the tricarboxylic acid (TCA) cycle intermediate succinic acid was increased in W but declined in C, while that of L-malic acid decreased in both soybean genotypes, but especially in C. The metabolites related to glycolysis, including fructose-6-phosphate, glyceric acid and pyruvic acid, decreased significantly in C young leaves (*P* < 0.05). However, in W young leaves, the levels of glucose-6-phosphate and pyruvic acid decreased, while those of fructose-6-phosphate and glyceric acid increased.

**Table 3:** Changes of LN stress on metabolite content in young leaves of two wild soybean varieties

Metabolite name		Relative concentration				Fold changes Log_2_^(LN/CK)^	
		C		W			
		CK	LN	CK	LN	C	W
Aliphatic amino acid	Valine	25.53 ± 2.6	5.57 ± 1.11	0.62 ± 0.04	0.54 ± 0.05	−2.20^**^	−0.19^**^
	Isoleucine	15.35 ± 0.21	1.95 ± 0.02	0.39 ± 0.01	0.11 ± 0.00	−2.98^**^	−1.83^**^
	Aspartic acid	14.25 ± 0.13	7.10 ± 0.05	5.30 ± 0.06	9.35 ± 0.01	−1.00	0.82^**^
	Asparagine	48.53 ± 0.19	0.28 ± 0.10	2.58 ± 0.67	0.60 ± 0.76	−7.46^**^	−2.09^**^
	L − homoserine	0.73 ± 0.05	0.13 ± 0.07	0.22 ± 0.00	0.08 ± 0.00	−2.54^**^	−1.56
	L − threonine	10.60 ± 0.20	1.17 ± 0.22	1.77 ± 0.03	1.82 ± 0.04	−2.79^**^	0.04
	Serine	47.67 ± 0.04	8.46 ± 0.02	0.19 ± 0.13	0.16 ± 0.25	−2.49^**^	−0.27
	L − proline	12.72 ± 0.67	3.47 ± 0.11	0.27 ± 0.00	0.21 ± 0.02	−1.87^**^	−0.40^*^
	Alanine	89.09 ± 0.15	40.72 ± 0.07	0.06 ± 0.04	0.07 ± 0.03	−1.13^**^	0.35
	Glycine	17.12 ± 6.96	2.93 ± 7.54	28.26 ± 0.02	50.61 ± 0.04	−2.55^**^	0.84^*^
	Glutamate	0.09 ± 0.00	0.03 ± 0.00	0.03 ± 0.00	0.03 ± 0.00	−1.86^*^	−0.31
Aromatic amino acid	Tyrosine	3.28 ± 0.11	0.47 ± 0.01	1.68 ± 0.30	1.11 ± 0.04	−2.80^**^	−0.60^**^
	Phenylalanine	4.26 ± 1.44	1.72 ± 0.54	11.31 ± 0.07	10.73 ± 0.02	−1.30^**^	−0.08
Non − protein nitrogen	GABA	5.35 ± 0.10	7.28 ± 0.21	0.52 ± 0.01	0.32 ± 0.03	0.44	−0.70^*^
	Ethanolamine	61.53 ± 1.84	24.49 ± 0.50	55.44 ± 0.02	86.12 ± 0.04	−1.33^**^	0.64^*^
	Mannosamine	0.19 ± 0.31	0.13 ± 0.04	11.32 ± 0.00	7.19 ± 0.01	−0.57^*^	−0.65
	Sphingosine	2.49 ± 0.14	2.07 ± 0.06	1.76 ± 0.30	2.34 ± 0.34	−0.27	0.41
Sugars and polyols	Sucrose	0.63 ± 0.08	2.08 ± 0.35	0.08 ± 0.07	0.07 ± 0.00	1.72^*^	−0.10
	Maltose	1.83 ± 4.23	2.27 ± 0.41	0.33 ± 0.01	0.28 ± 0.02	0.31^**^	−0.21
	Maltotriose	0.05 ± 0.02	0.22 ± 0.04	0.93 ± 0.02	0.96 ± 0.03	2.14^**^	0.04
	Melezitose	0.05 ± 0.27	0.28 ± 0.02	28.10 ± 0.38	20.39 ± 0.02	2.54^**^	−0.46
	Threitol	0.47 ± 0.02	0.35 ± 0.03	2.57 ± 0.20	1.72 ± 0.08	−0.44^**^	−0.58
	Galactinol	0.13 ± 0.02	0.05 ± 0.00	0.71 ± 0.01	0.52 ± 0.02	−1.34^*^	−0.47
	Glycerol	12.81 ± 0.07	7.79 ± 0.03	1.91 ± 0.28	1.56 ± 0.27	−0.72^*^	−0.29
	Myo − inositol	55.75 ± 0.09	28.6 ± 0.09	0.31 ± 0.02	0.22 ± 0.03	−0.96^*^	−0.48
	L − threose	0.42 ± 0.01	0.13 ± 0.00	54.13 ± 0.01	60.66 ± 0.16	−1.68^*^	0.16
	Xylose	0.71 ± 0.01	0.91 ± 0.01	0.09 ± 0.02	0.11 ± 0.01	0.35**	0.29
	Glucose−6 − phosphate	0.07 ± 0.00	0.20 ± 0.14	306.86 ± 0.34	216.33 ± 0.17	1.49**	−0.50*
	Fructose-6 − biphosphate	0.48 ± 0.02	0.14 ± 0.01	0.08 ± 0.00	0.28 ± 0.01	−1.71*	1.84**
﻿Organic acid	Glyceric acid	14.14 ± 1.77	6.57 ± 1.27	0.33 ± 0.55	0.43 ± 0.09	−1.11**	0.36
	Pyruvic acid	5.89 ± 1.52	2.33 ± 1.10	0.20 ± 0.55	0.13 ± 0.30	−1.34**	−0.64*
	succinic acid	8.49 ± 0.08	8.19 ± 0.41	0.25 ± 0.02	0.32 ± 0.02	−0.05	0.37*
	Fumaric acid	77.42 ± 0.09	111.54 ± 0.04	4.50 ± 0.25	2.40 ± 0.16	0.53	−0.91*
	L − malic acid	0.18 ± 0.32	0.07 ± 0.02	4.04 ± 0.18	3.17 ± 0.15	−1.36**	−0.35
	Hydroxypropionic acid	0.33 ± 0.29	0.27 ± 0.03	0.02 ± 1.15	0.02 ± 0.58	−0.25	−0.02
	Oxalic acid	0.37 ± 2.73	0.28 ± 0.00	0.97 ± 1.79	0.78 ± 0.03	−0.42*	−0.31
	Itaconic acid	4.36 ± 2.95	3.74 ± 0.00	2.90 ± 0.03	3.91 ± 1.79	−0.22	0.43
	Malonate	0.29 ± 9.64	0.08 ± 0.30	4.47 ± 0.08	5.48 ± 0.51	−1.79**	0.30
	Maleic acid	9.83 ± 0.15	11.31 ± 0.15	0.08 ± 0.03	0.18 ± 0.03	0.20	1.11*
	Gluconic acid	0.56 ± 0.39	0.23 ± 0.57	0.56 ± 0.07	0.78 ± 0.09	−1.31**	0.49**
	Nicotinic acid	2.45 ± 0.51	1.73 ± 0.11	4.95 ± 0.58	10.38 ± 0.40	−0.50	1.07**
	Threonic acid	2.40 ± 0.34	2.02 ± 0.19	2.23 ± 0.83	0.72 ± 0.00	−0.25	−1.63*
	Galactonic acid	1.92 ± 0.62	1.38 ± 0.63	0.83 ± 0.00	0.51 ± 0.10	−0.47*	−0.70**
	Glucoheptonic acid	0.78 ± 0.26	0.54 ± 0.02	16.23 ± 0.31	8.81 ± 0.41	−0.52	−0.88
Fatty acids	Butyrate	0.23 ± 0.05	0.12 ± 0.00	2.73 ± 0.01	1.60 ± 0.40	−0.93**	− 0.77
	Stearic acid	0.66 ± 0.15	0.29 ± 0.03	0.24 ± 0.01	0.11 ± 0.03	−1.21*	−1.18*
	Palmitic acid	3.60 ± 0.55	1.28 ± 0.20	42.32 ± 1.77	40.01 ± 1.53	−1.49*	−0.08
	Linolenic acid	2.55 ± 0.72	1.20 ± 0.17	0.17 ± 0.05	0.14 ± 0.02	−1.09**	−0.29
	Linoleic acid	0.14 ± 0.01	0.04 ± 0.03	4.13 ± 0.09	2.72 ± 0.13	−1.71**	−0.60**
Phenylpropanoids	Neohesperidin	0.37 ± 0.00	0.23 ± 0.00	11.03 ± 0.05	7.74 ± 0.21	−0.69*	− 0.51*
	Salicylic acid	3.97 ± 0.03	1.25 ± 0.13	0.13 ± 0.34	0.18 ± 0.13	−1.66**	0.52
	Prunin	0.24 ± 0.09	0.18 ± 0.04	8.47 ± 1.06	13.32 ± 0.36	−0.39	0.65**
	Ferulic acid	2.25 ± 0.03	1.09 ± 0.04	13.46 ± 0.03	36.23 ± 0.13	−1.04**	1.43**
	Caffeic acid	0.02 ± 0.24	0.01 ± 0.24	1.30 ± 0.00	0.36 ± 0.00	−0.55	−1.86*

Relative concentrations and standard deviation were increased by a factor of 100 times in each treatment. Values were presented as the mean ± standard error of four biological replicates. *C* Cultivar soybean, *W* Wild soybean, *CK* Control treatment, *LN* Low−N stress; * and ** indicate significant (*P* < 0.05) and highly significant (*P* < 0.01) differences, respectively

The contents of differential amino acids, including the aliphatic amino acids isoleucine, aspartic acid, asparagine, L-homoserine, L-threonine, serine, proline, alanine, glycine and glutamate, and ﻿the aromatic amino acids tyrosine and phenylalanine, significantly decreased in C young leaves during LN stress compared with the CK (*P* < 0.05). The non-protein N, such as ethanolamine, monoamine and sphingosine, had the same trend. Under the same treatment, L-threonine, alanine, glycine, ethanolamine (aliphatic amino acid) and sphingosine (nonprotein N) levels increased in W young leaves, while the other amino acids and non-protein N decreased in W young leaves. However, the ranges of the decreases were less than those in C. In C young leaves, the contents of polyols, like threitol, galactinol, glycerol, myo-inositol and L-threose (monosaccharide), decreased significantly (*P* < 0.05), while the contents of xylose (monosaccharide), sucrose, maltose (disaccharides), maltotriose and melezitose (trisaccharide) increased. The polyols and carbohydrate metabolites did not significantly change in W (*P* > 0.05). In W young leaves, the levels of organic acids, including itaconic, methylmalonic, maleic, gluconic and nicotinic acids, increased in response to LN stress. The contents of threonic acid, galactonic acid, glucoheptonic acid, 4-hydroxybutyrate, hydroxypropionic acid and oxalic acid decreased in both soybean genotype, but 4-hydroxybutyrate, hydroxypropionic acid and oxalic acid decreased more significantly in C young leaves (P < 0.05). The levels of the stearic, palmitic, linolenic and linoleic fatty acids decreased in both soybean genotypes, but especially in C young leaves. The contents of phenylpropanoids, including salicylic acid, prunin and ferulic acid, increased in W young leaves, while the opposite trend occurred in C young leaves. Neohesperidin and caffeic acid levels decreased in the young leaves of both soybean genotypes.

In the old leaves of W and C, the levels of 64 metabolites were independently calculated and compared (Table 4; Fig. 4b). In W old leaves, the intermediates of the TCA cycle, including fumaric, malic, citramalic, succinic and succinic acids, increased, while the opposite trend occurred in C old leaves. The contents of glycolysis-related metabolites increased in W old leaves. Compared with the CK, the levels of galactose, fructose-6-phosphate, glucose-6-phosphate, lactic acid and galactonic acid increased but those of glyceric and pyruvic acids decreased in C old leaves. The amino acids contents all increased in W old leaves under LN-stress conditions but did not significantly change in C old leaves (*P* > 0.05). The contents of non-protein N in W old leaves also increased, while 4-aminobutyric acid and maleimide decreased in C. The contents of polyols, including monopalmitin, myo-inositol, palatinitol, arabitol, threitol and erythritol, increased in the old leaves of both soybean genotypes, but especially in W. Sucrose decreased in the both genotypes old leaves, while maltose, melezitose, L-threose and fucose levels increased in W old leaves under LN-stress conditions. The contents of organic acids, including ketoisocaproic, 2-deoxytetronic, oxalic, propionic, gluconic and glucoheptonic acids, increased in W and C old leaves, but the increase was more significant in the former. The contents of butyrate and maleic acid decreased in C old leaves and increased in those of W. Among the fatty acids, stearic, linoleic, palmitic, linolenic and isobutyric acid contents increased, while that of butyric acid decreased, in both soybean genotypes’ old leaves. The contents of neohesperidin, salicylic acid, trans-cinnamate acid and naringin increased significantly in W and C old leaves (*P* < 0.05), while the contents of ferulic acid and caffeic acid increased in C old leaves but declined in W old leaves.

**Table 4:** Changes of LN stress on metabolites content in old leaves of two wild soybean varieties

Metabolite name		Relative concentration				Fold changes Log_2_^(LN/CK)^	
		C		W			
		CK	LN	CK	LN	C	W
Aliphatic amino acid	Asparagine	0.51 ± 0.06	0.06 ± 0.02	0.06 ± 0.01	0.22 ± 0.05	−3.20^**^	1.95^*^
	Aspartic acid	1.53 ± 0.33	0.79 ± 0.24	0.07 ± 0.04	0.33 ± 0.09	−0.95	2.28^*^
	Threonine	1.43 ± 0.03	1.24 ± 0.15	0.02 ± 0.00	0.03 ± 0.01	−0.20	0.97^*^
	Cycloleucine	0.85 ± 0.06	1.31 ± 0.55	30.89 ± 5.31	44.38 ± 8.78	0.63	0.52
	Isoleucine	5.58 ± 0.20	2.58 ± 0.19	0.38 ± 0.01	1.33 ± 0.25	−1.11	1.79^**^
	L − proline	2.46 ± 1.02	5.06 ± 0.68	0.11 ± 0.01	0.81 ± 0.18	1.04	2.85^**^
	L − homoserine	0.29 ± 0.05	0.33 ± 0.02	1.04 ± 0.21	2.42 ± 0.26	0.19	1.22^**^
	Valine	5.63 ± 0.87	4.42 ± 0.09	37.13 ± 3.86	47.46 ± 5.60	−0.35^*^	0.35
	Alanine	2.46 ± 0.32	1.61 ± 0.52	0.09 ± 0.01	0.26 ± 0.03	−0.61	1.57^**^
	Glycine	3.51 ± 0.54	2.62 ± 0.46	1.07 ± 0.15	1.27 ± 0.30	−0.42^*^	0.25
	Serine	8.50 ± 0.84	12.51 ± 2.47	0.01 ± 0.00	0.06 ± 0.01	0.56	2.32^*^
	Sarcosine	1.59 ± 0.17	2.07 ± 0.23	0.81 ± 0.15	2.97 ± 0.63	0.38	1.88^*^
Aromatic amino acid	Phenylalanine	0.71 ± 0.21	0.49 ± 0.04	0.08 ± 0.03	0.17 ± 0.02	−0.53	1.01^*^
Non − protein nitrogen	Galactosamine	0.08 ± 0.01	0.10 ± 0.00	9.79 ± 4.25	17.95 ± 3.79	0.26	0.87
	GABA	8.34 ± 0.78	6.38 ± 1.42	0.10 ± 0.02	0.19 ± 0.02	−0.39	0.92^*^
	Maleimide	0.93 ± 0.08	0.77 ± 0.18	0.23 ± 0.01	0.31 ± 1.10	−0.27	0.43^*^
	Mannosamine	0.83 ± 0.09	1.06 ± 0.08	3.67 ± 0.66	10.39 ± 0.63	0.36	1.50^**^
	Ethanolamine	19.62 ± 0.57	20.71 ± 4.45	30.85 ± 6.18	61.33 ± 6.31	0.08	0.99^*^
Sugars and polyols	Glycerol	14.12 ± 0.18	11.49 ± 0.27	0.10 ± 0.01	0.20 ± 0.00	−0.30	0.95^**^
	Monopalmitin	0.04 ± 0.01	0.16 ± 0.05	25.16 ± 8.47	106.06 ± 10.98	1.83	2.08
	Myo − inositol	19.77 ± 0.48	28.56 ± 1.03	0.60 ± 0.07	1.48 ± 0.41	0.53^**^	1.31^**^
	Palatinitol	0.04 ± 0.00	0.06 ± 0.00	0.29 ± 0.06	0.52 ± 0.11	0.78^**^	0.85
	Arabitol	1.46 ± 0.14	1.57 ± 0.06	0.12 ± 0.01	0.57 ± 0.10	0.10	2.23^**^
	Threitol	0.84 ± 0.23	1.25 ± 0.14	0.17 ± 0.01	0.37 ± 0.06	0.57	1.15^*^
	Erythritol	0.08 ± 0.02	0.20 ± 0.03	1.23 ± 0.25	4.26 ± 0.15	1.24^**^	1.79^*^
	Sucrose	36.97 ± 1.51	29.08 ± 1.92	30.81 ± 9.34	2.64 ± 0.02	−0.35^**^	−3.54^*^
	Maltose	3.03 ± 0.42	2.37 ± 0.32	0.12 ± 0.01	0.22 ± 0.05	−0.36	0.91
	Melezitose	0.24 ± 0.11	0.23 ± 0.02	0.004 ± 0.00	0.03 ± 0.00	−0.11	2.77^**^
	L − threose	0.12 ± 0.03	0.12 ± 0.01	4.15 ± 0.23	4.96 ± 0.75	−0.04	0.26
	Fucose	0.17 ± 0.06	0.10 ± 0.00	0.08 ± 0.00	0.12 ± 0.01	−0.81	0.68^**^
	Xylose	0.22 ± 0.03	0.37 ± 0.03	6.36 ± 0.17	10.71 ± 2.81	0.75^*^	0.75
	Galactose	0.87 ± 0.20	1.70 ± 0.15	0.05 ± 0.01	0.08 ± 0.01	0.96^*^	0.53
	Fructose-6 − biphosphate	0.06 ± 0.01	0.22 ± 0.04	0.06 ± 0.00	0.15 ± 0.01	1.83^**^	1.25^**^
	Glucose−6 − phosphate	0.03 ± 0.00	0.14 ± 0.04	0.95 ± 0.15	1.55 ± 0.22	2.49^*^	0.70
Organic acid	Lactic acid	2.17 ± 0.21	4.79 ± 0.91	0.69 ± 0.09	0.93 ± 0.17	1.15^*^	0.44
	Galactonic acid	0.63 ± 0.08	2.08 ± 0.25	1.05 ± 0.35	1.42 ± 0.14	1.72^**^	0.44
	Glyceric acid	8.33 ± 0.94	8.12 ± 2.57	9.87 ± 0.91	15.71 ± 2.56	−0.04	0.67^*^
	Pyruvic acid	7.98 ± 0.17	2.84 ± 0.71	0.34 ± 0.08	0.85 ± 0.11	−1.49^**^	1.31^*^
	Fumaric acid	44.89 ± 4.92	7.64 ± 1.23	0.06 ± 0.02	0.07 ± 0.01	−2.55^**^	0.11
	Malic acid	0.16 ± 0.01	0.11 ± 0.01	3.39 ± 0.57	5.29 ± 1.30	−0.50^*^	0.64
	Citramalic acid	0.35 ± 0.06	0.23 ± 0.02	0.16 ± 0.00	0.26 ± 0.04	−0.62	0.71^*^
	Succinic acid	7.73 ± 0.72	7.15 ± 1.28	6.33 ± 1.30	10.06 ± 0.74	−0.11	0.67^*^
	Ketoisocaproic acid	0.02 ± 0.00	0.03 ± 0.00	11.15 ± 0.46	22.50 ± 0.38	0.55^**^	1.01^**^
	2 − Deoxytetronic acid	18.32 ± 3.67	30.44 ± 2.30	3.47 ± 0.61	9.71 ± 1.54	0.73	1.49
	Oxalic acid	0.19 ± 0.03	0.24 ± 0.04	0.12 ± 0.01	0.26 ± 0.04	0.71	2.26^*^
	Propionic acid	0.14 ± 0.03	0.17 ± 0.01	0.83 ± 0.20	3.03 ± 0.34	0.34	1.13^**^
	Maleic acid	14.74 ± 1.66	7.75 ± 1.14	1.73 ± 0.20	9.20 ± 3.54	0.28^*^	1.86
	Gluconic acid	0.11 ± 0.01	0.12 ± 0.02	0.10 ± 1.03	0.34 ± 0.09	−1.10	0.58^*^
	Glucoheptonic acid	1.98 ± 0.13	2.64 ± 0.20	1.71 ± 0.49	3.57 ± 0.71	−0.93	2.41
Fatty acids	Butyrate	0.31 ± 0.01	0.15 ± 0.07	25.66 ± 1.03	38.35 ± 2.81	0.13	1.83^*^
	Isobutyric acid	0.26 ± 0.05	0.43 ± 0.04	2.53 ± 0.38	12.14 ± 3.61	0.41	1.06
	Stearic acid	0.16 ± 0.01	0.42 ± 0.07	1.92 ± 0.10	3.50 ± 0.53	1.43^*^	0.87^*^
	Linoleic acid	0.01 ± 0.00	0.02 ± 0.00	8.30 ± 2.49	22.03 ± 0.87	0.83^*^	1.41^**^
	Linolenic acid	0.34 ± 0.03	1.14 ± 0.16	3.19 ± 0.40	2.49 ± 0.33	1.75^**^	−0.36
	Palmitic acid	0.55 ± 0.04	1.64 ± 0.22	0.27 ± 0.02	0.82 ± 0.26	1.56^**^	1.59
	Butyric acid	0.31 ± 0.22	0.14 ± 0.01	0.95 ± 0.10	0.67 ± 0.04	−1.09	−0.52^*^
Phenylpropanoids	Neohesperidin	0.81 ± 0.11	2.14 ± 0.45	0.17 ± 0.03	1.27 ± 0.24	1.40^*^	2.90^**^
	Salicylic acid	1.23 ± 0.00	1.60 ± 0.17	2.28 ± 0.24	7.70 ± 1.42	0.38	1.76^**^
	Trans − cinnamate	0.36 ± 0.03	1.08 ± 0.20	1.43 ± 0.11	4.59 ± 0.74	1.59^*^	1.68^**^
	Naringin	0.01 ± 0.00	0.06 ± 0.02	0.78 ± 0.24	1.81 ± 0.59	3.04^*^	1.21
	Ferulic acid	0.81 ± 0.08	1.16 ± 0.05	0.47 ± 0.04	0.31 ± 0.03	0.52^*^	−0.61^**^
	Caffeic acid	0.01 ± 0.00	0.02 ± 0.00	6.48 ± 0.68	5.12 ± 0.54	1.16	−0.34
	Prunin	0.29 ± 0.02	0.19 ± 0.07	2.41 ± 0.51	5.61 ± 0.19	−0.61^*^	1.22^*^

Relative concentrations and standard deviation were increased by a factor of 100 times in each treatment. Values were presented as the mean ± standard error of four biological replicates. *C* Cultivar soybean, *W* Wild soybean, *CK* Control treatment, *LN* Low−N stress; * and ** indicate significant (*P* < 0.05) and highly significant (*P* < 0.01) differences, respectively

## Discussion

LN stress can retard plant development [22, 23]. Our biomass results largely confirmed previously reports in which W had a greater LN tolerance [24]. We then distinguished different responses between young and old leaves under LN-stress conditions, providing new insights into the strategies of W resistance to LN stress. The total carbon content (%) and total nitrogen content (%) result showed C had a worse carbon deficiency in C young leaves than W young leaves. While in W young leaves total carbon content (%) and total nitrogen content (%) maintained a relatively normal state, probable cause may be the transport of N from the old leaves, as the N content in the old leaves is significantly reduced [17]. Chlorophylls contents, as the nitrogen statue marker in leaves, decreased less in wild soybean young and old leaves than that in cultivated soybean. Chloroplast proteins proteolysis in senescence leaves and the liberated amino acids can be exported to growing parts of the plant [14]. The analyses of gas-exchange parameters and chlorophyll contents changes provide insights into the photosynthetic and nitrogen states of plants under different conditions [24]. Under LN-stress conditions, compared with CK, *P*_N_ in W old and young leaves decreased less than that in C. The decrease in *P*_N_ was evaluated in terms of non-stomatal factors, which include biochemical and structural processes [25, 26]. *E* in W young leaves increased under LN-stress conditions, and the increase in transpiration could promote the transport and transportation of ions [8]. There are balances and dependencies among the various elements in crops, and the lack of certain elements often affects the transport, accumulation and metabolism of other nutrients [27]. Compared with CK, the Fe^3+^, Mn^2+^ and H_2_PO_4_^−^ contents in W young and old leaves increased significantly more than in C under LN-stress conditions, and the levels of these ions were positively correlated with N metabolism [28–30]. The increase of B in W young leaves under LN-stress conditions was much greater than that in C. The B^3+^ content could enhance photosynthesis and promote vascular bundle development in legumes; therefore, rhizobia could obtain sufficient carbohydrate supplies to enhance the N fixation capability [31]. The NO_3_^−^ content decreased less in W than in C, and also decreased less in young leaves than in old leaves. This corroborates previous results in which NRT1.7 was indicated to regulate NO_3_^−^ transport out of older leaves and into younger leaves [17]. Our study indicated that W young leaves could maintain a relatively stable gas-exchange coefficient, while W young and old leaves increased their transport and remobilization levels of beneficial ions, which is important to sustain vigorous growth during N deficiency.

Combining with the previous research, short-term relative LN treatment of this strength had no significant effect on soybean root nodules development [24]. Therefore, we considered that the response of two soybean genotypes to LN stress was mainly in the metabolic level. Carbon and nitrogen metabolism are tightly coupled in different living organisms, which is essential for every biological system, since all major cellular components, including genetic materials, proteins, pigments, energy carrier molecules, etc., are derived from these activities. Under stress conditions, different regulatory levels exist in cells to maintain the properly balanced metabolism ratio between carbon and N that is necessary to avoid metabolic inefficiencies [32, 33]. N may acted as the signal to initiate coordinated changes in carbon and N metabolisms and organic acid production [33]. Adverse growth conditions induce the accumulation of polyols. The function of polyols as “stress metabolites” and correlated expression patterns have been observed across environmental gradients [34]. During LN stress, compared with CK, there was less inhibition of sugar and polyol metabolism in W young leaves than in C. However, sugar and polyol metabolism in W old leaves increased insignificantly, especially polyol metabolism. Although hydroxyls produced by nitrate reduction decreased during LN stress, the frequency of electron carriers in the electron transport chain decreased, resulting in a significant increase in reactive oxygen species [34]. Polyols are major constituents of plant soluble components, and they are important substances involved in intracellular osmotic regulation and in enhancing resistance to reactive oxygen species [35]. Moreover, up to 30% of the gross primary production was thought to proceed through polyols in place of carbohydrates [36]. The enhancement of polyol metabolism, including the production of monopalmitin, myo-inositol, palatinitol, arabitol, threitol and erythritol, mainly occurred in W old leaves under LN-stress conditions. In contrast to carbohydrates, polyols lack aldehyde and ketone functional groups, making them well suited to transport entities and storage molecules [37]. Thus, ﻿they function to transport carbon skeletons and energy between source and sink organs [36]. The strategy of enhancing polyols metabolism and transport polyols from old leaves to young leaves could effectively improve the LN tolerance of W.

The results showed that saturated fatty acid and organic acid contents in W old leaves increased which may be also adaptive strategies of W under adversity [38]. Organic acid metabolism not only provides carbon skeletons during N assimilation but also has potential roles in osmotic regulation, cation balance, nutrient deficiency-related coping mechanisms and plant–microbe interactions at the root–soil interface [38]. Compared with CK, the organic acid metabolism, producing itaconic, gluconic and nicotinic acids, increased in W young leaves under the LN-stress conditions. Additionally, in W old leaves, the metabolisms of ketoisocaproic, 2-deoxytetronic, oxalic, propionic, gluconic and glucoheptonic acids showed the same trend. The accumulation of organic acids can improve soil acidity and increase the availability of rhizospheric soil [38]. Another probable significance of organic acid accumulation is their participation in balancing the charges formed during the extensive metabolism of anions, such as NO_3_^−^ [39]. The rate of N uptake by soybean roots may increase when stimulated by organic acids [40].

Phenylpropanoid biosynthesis in plants engenders a vast variety of aromatic metabolites that are critical for their growth, development and environmental adaptability [41]. All phenylpropanoids, such as flavonoids, lignins and alkaloids, are derived from trans-cinnamic acid, which is formed from phenylalanine by the action of phenylalanine ammonia-lyase [42]. Here, the phenylpropanoid biosynthetic pathway was significantly active in W young and old leaves under LN-stress conditions. The increased metabolisms of phenylalanine and trans-cinnamic acid ensured an adequate supply of substrates to produced beneficial secondary metabolites. The positive correlation between flavonoid metabolic levels and antioxidant properties has been reported, and flavonoids can promote plant-microbe interactions and enhance root colonization by microbes [43, 44]. Moreover, salicylic acid may be part of the signaling process that results in systemic acquired resistance [45].

The metabolism of carbon and N also involves energy metabolism. Compared with CK, glycolysis in W young and old leaves was maintained at a relatively stable state under LN-stress conditions. In C ​​old leaves, the first stage of glycolysis (energy-consuming process) was enhanced, but the second stage (energy-producing) was decreased. Prophase studies in functional leaves revealed that soybean has a glycolysis-enhancing strategy to adapt to abiotic stress [13]. Although enhanced glycolysis levels could compensate for lower ATP yields, limited energy would be generated owing to the incomplete oxidation of organic matter, and there was a risk of depleting the respiratory substrate, which is uneconomical for plant growth [46]. The TCA cycle is the most important source of energy for cells. In W young and old leaves, the TCA cycle is significantly enhanced, which ensured that W could maintain a relatively stable supply of material and energy metabolism, resulting in the production of a steady energy supply during LN stress. However, the opposite trend occurred in C, and the production of important TCA cycle intermediates, including succinic acid and malic acid, was significantly inhibited in C young leaves. However, half of the TCA cycle intermediates represent the origins of pathways leading to important metabolites, such as fatty acids, amino acids and porphyrins [47]. Therefore, the energy metabolism-related strategies of W young and old leaves had obvious advantages compared with those of C under LN-stress conditions.

Amino acid metabolism is of crucial importance in N metabolism because it influences plant cell behavior in a myriad of ways [48]. The metabolism of amino acids in W young leaves was relatively stable, but the metabolism of amino acids in C young leaves was significantly inhibited. In W old leaves, amino acid metabolism was significantly enhanced, while it showed no significant change in C old leaves. In addition to their roles as protein constituents, amino acids are also involved in plant growth and development, osmotic adjustment, anti-oxidation, intracellular pH control, metabolic energy production and resistance to both abiotic and biotic stresses [49]. The enhanced amino acids metabolism in W old leaves resulted in increased contents of nitrogenous compounds. This strategy might provide a material basis for nitrogen and nitrogen transport among old and young leaves. On the contrary, N metabolism in C old leaves underwent no significant change, but the amino acid contents were significantly decreased in C young leaves under LN-stress conditions, compared with the CK. This further confirmed that the LN resistance in C was correlated with the inadequate N transport and reutilization capacities from young to old leaves. Proline is a major organic osmolyte that accumulates in some plant species in response to environmental stresses [50]. Proline metabolism was enhanced in W old leaves, which improved the LN tolerance. The reverse regulation of the pathways that compete with amino acid synthesis in plants could balance the C and N metabolism in plants [49]. When the energy supply was sufficient, including the high light and high-concentration carbohydrate, GS and GOGAT cycle could be activated and will promote N assimilation into glutamic acid metabolism. On the contrary, GS and Fd-GOGAT were inhibited, while AS was activated, and N assimilation proceeded towards asparagine metabolism [51]. The reverse regulation of these competing pathways can balance Carbon and Nitrogen metabolism in plants [52, 53]. In plants, the aspartic acid metabolism is highly important because it culminates with the synthesis of several essential amino acids, such as L − homoserine, L − threonine, Alanine, lysine, threonine and isoleucine [54]. Asparagine plays a central role in N transport and storage in plants owing to its high N/carbon ratio and stability. In older leaves nitrogen is not required for growth, and previous studies have shown that transpirationally derived asparagine in older leaves is re-exported to the apex [54, 55]. W old leaves may have increased aspartic acid metabolism to maintain the stability and balance of N nutrition from old leaves to young leaves. GABA is involved in the temporary storage of N and can be an anaplerotic compound by providing TCA cycle intermediates during stress responses [56]. Additionally, metabolome and transcriptome studies indicate that GABA might play a role in coordinating the carbon–N balance and even mediate a starvation response in plant cells [57, 58]. The accumulation of GABA in W old leaves ensured efficient N reuse, which is an essential process to maintain the carbon and N balance [54]. Oxidative stress induced by abiotic stress enhances protein catabolism and results in increased amino acids levels [19, 59]. The current challenge is to elucidate the diverse mechanisms of amino acid enrichment.

## Conclusion

The survival and growth of wild soybean under LN-stress conditions relied on physiological adjustments and metabolic changes in plant. Compared with cultivated soybean, wild soybean young leaves maintained relatively stable assimilative capacity and increased the cyclic utilization of beneficial ions. The relative normal growth of wild soybean young leaves under the low nitrogen condition mainly owing to a significant enhancement of nitrogen metabolism in old leaves, especially aspartic acid, proline and GABA metabolism. Therefore, the increased of key amino acids and nonprotein nitrogen provided a material basis for N storage, transportation and reutilization from old leaves to young leaves. Meanwhile, intensifying phenylpropanoid metabolic pathway in old young and old leaves had a positive effect in improving the low nitrogen resistance of wild soybean. Besides, consolidating the TCA cycle to ensure the energy supply, and accumulating more polyols and organic acids in old and young leaves, which not only alleviated the carbohydrate deficiency in developing young leaves, but also improved antioxidant and N fixation capacities. The results provide a foundation for further functional studies to explore metabolite regulation during abiotic-stress resistance in plants.

## Methods

### Plant materials and growth conditions

The experimental materials, seeds of wild soybean (W; ‘Huinan06116’) and cultivar soybean (C; ‘Jinong24’), were provided by Jilin Academy of Agriculture Science, China. The seedlings were grown arranged in 14-cm diameter pots with a bottom hole (2 cm in diameter) with clean sand. The seedlings were grown in an outdoor experimental field at Northeast Normal University, Changchun, Jilin. The average growth temperatures were 18.5 ± 1.5 °C and 26 ± 2 °C during the night and day, respectively, and the relative humidity was 60 ± 5%. The seedlings were germinated by irrigation with water.

### Stress treatments

W and C were both randomly divided into two groups, with eight pots each: control and LN-treated. Four pots were used for measuring photosynthetic parameters and ion content, and the remaining four pots for metabolomics analyses in each group. The LN treatment was initiated when the seedlings’ third leaves had grown. In the LN-treated group, W and C seeds were placed in 1/4-strength modified Hoagland’s solution for two weeks, and CK was cultivated under normal conditions (1× Hoagland’s solution [24].

### Photosynthetic indices measurements

Two weeks after the stress treatment, choosing the first two blade from the top and the first two blade from the bottom of the shooting as the young leaves and the old leaves in four pots receiving the same treatment in each pot. Three young and old leaves were selected in each pot, and three data points were recorded per leaf, for a total of 72 data points per parameter. The photosynthetic rate (*P*_N_), stomatal conductance (*g*_s_), intercellular CO_2_ concentration (*C*_i_ /*Ca*) and transpiration rate (*E*) values of leaves were determined using a LI-6400 portable open flow gas exchange system (LI-COR, USA) at 11:00 AM. The concentration of CO_2_ in the atmosphere, effective photosynthetic radiation, air temperature and humidity were 380 ± 5 cm^3·^m^− 1^, 1200 ± 50 μmol·m^− 2^·s^− 1^, 24 °C and 50%, respectively [60] .

Dry leaf samples (30 mg) were dipped into 10 ml of 80% acetone: anhydrous ethanol mixture (1:1) to extract the photosynthetic pigments in darkness at room temperature until the leaves became white. Each sample was repeated three times. Spectrophotometric (SpectrUV-754, Shanghai Accurate Scientific Instrument Co.) determinations at 440, 645 and 663 nm for each sample were performed three times using the formulae of Holm (1954).

### Growth indices measurements

After the soybean plants were harvested, plant heights, root lengths, aboveground fresh weight (Up FW), underground FW (Under FW), aboveground dry weight (Up DW), and underground dry weight (Under DW) were measured [8].

### Measurement of ion content

Dry 0.05 g samples were treated with 4 mL of deionized water at 100 °C for 40 min, and then centrifuged at 3000 g for 15 min, the supernatant was collected, and the course was repeated twice, with extracts made up to 15 mL. Unified supernatants were used to determine SO_4_^2−^, NO_3_^−^, H_2_PO_4_^−^, Cl^−^ and C_2_O_4_^2−^ concentrations by ion chromatography (DX-300 ion chromatographic system, AS4A-SC chromatographic column, CDM-II electrical conductivity detector, mobile phase: Na2CO3/NaHCO3 = 1.7/1.8 mM, Dionex, Sunnyvale, CA, USA). An atomic absorption spectrophotometer (Super 990F, Beijing Purkinje General Instrument Co. Ltd., Beijing, China) was used to determine the concentrations of Na^+^, K^+^, Ca^2+^, Mg^2+^, Fe^3+^, B^3+^, P^5+^, Zn^2+^ and Mn^2+^ [60].

### Metabolite extraction and profiling analysis

Samples (50 mg) were transferred to 1.5 mL EP tubes (Eppendorf Micro Test Tubes, Eppendorf China Limited, Shanghai City, China), then added internal standard that consisted of 0.5 mL of extraction liquid (V (methanol): V (chloroform) = 3:1) and 60 μL of ribitol (0.2 mg mL − 1stock in H_2_O). Centrifuged the simples for 10 min at 12,000 g, at 4 °C (Tabletop Low-Speed Centrifuge L-500, Hunan Saite Xiangyi Centrifuge Instrument Co., Ltd., Hunan City, China), Then mixing thoroughly. 0.4 mL of the supernatant was transferred into a 2 mL GC-MS glass vial as a new sample. Exactly 80 μL of methoxyamination reagent (20 mg mL^− 1^ methoxylamine hydrochloride in pyridine) was added to the new samples. Then vortexed them for 10 s, dried in a vacuum concentrator, and placed in an oven (MKX-J1–10, Qingdao Makewave Microwave Technology Co. Ltd., Qingdao, China) adjusted to 37 °C for 2 h. Finally, 0.1 mL of the BSTFA reagent (1% TMCS, v/v) was added into samples and they were oscillated at 70 °C for 1 h. After the simples temperature fell to room temperature, the GC–MS analysis was performed using an Agilent 7890 gas chromatograph system coupled to a Pegasus HT time-of-flight mass spectrometer (NYSE: A, Beijing City, China). A 1 μL of aliquot of the analyte was injected in splitless mode. Helium was used as the carrier gas with a flow rate of 20 mL min^− 1^ and the front inlet purge flow was 3 mL min^− 1^. The column temperature was maintained at 50 °C for the first 1 min and then was increased at a rate of 10 °C min^− 1^ until it reached 330 °C. The temperature was kept at 330 °C for 5 min. Ionization in the injection, transfer line, and ion source at 280, 280, and 220 °C, respectively, was coupled with electron energy of − 70 eV. Mass spectra data were recorded in the 85–650 m z^− 1^ range at a rate of 20 spectra [61, 62].

### Data processing and multivariate data analysis

The data were pre-processed by the manufacturer’s ChromaTOF software (versions 2.12, 2.22, 3.34; LECO, St. Joseph, MI, USA) [62]. The metabolites were identified by searching the commercial EI-MS and the FiehnLib libraries [63]. At least 80% of missing values were removed. These missing values were replaced with a small value, which was half of the minimum positive value in the original data. Then, the data were filtered using the IQR. In addition, the total mass of the signal integration area was normalized for each sample. Then the major metabolites were analyzed using Student’s T test (*p* < 0.05) and their similarity values if they were more than 500. Next, the normalized data were fed into the SIMCA-P 13.0 software package (Umetrics, Umea, Sweden) for PCA, PLS-DA, OPLS-DA. Subsequently, the metabolic pathway was constructed according to KEGG (http-://www.genome.jp/kegg/) and the pathway was analyzed using the MetaboAnalyst website (http://http://www.metaboanalyst.ca/), which was based on the change in metabolite concentration compared to the corresponding controls [64, 65].

## Additional files

Additional files 1: The growth performances in young and old leaves of two soybean varieties under LN stress. C, cultivated soybean; W, wild soybean; CK, control treatment; LN, low nitrogen stress; Up FW, aboveground fresh weight; Up DW, aboveground dry weight; Under FW, underground fresh weight; Under DW, underground dry weight. * and ** indicate significant (*P* < 0.05) and highly significant (*P* < 0.01) differences, respectively. (DOCX 13 kb)

Additional file 2: The contributions of ions among young and old leaves of W and C seedlings to the first principal component (PC1) and the second principal component (PC2). C, cultivar soybean; W, wild soybean; CK, control treatment; LN, low-nitrogen stress (XLSX 12 kb)

Additional file 3: Total ion current chromatograms of two genotypes soybean seedling leaves extracts obtained from GC-MS. A: W-YL-CK; B: W-YL-LN; C: W-OL-CK; D: W-OL-LN; E: C-YL-CK; F: C-YL-LN; G: C-OL-CK; H: W-OL-LN. (DOCX 1139 kb)

Additional file 4: The contributions of metabolites among young leaves of W and C seedlings to the first principal component (PC1) and the second principal component (PC2). C, cultivar soybean; W, wild soybean; CK, control treatment; LN, low-nitrogen stress (XLSX 14 kb)

Additional file 5: The contributions of metabolites among old leaves of W and C seedlings to the first principal component (PC1) and the second principal component (PC2). C, cultivar soybean; W, wild soybean; CK, control treatment; LN, low-nitrogen stress (XLSX 15 kb)

## Data Availability

The datasets generated and analyzed during the current study are available from the corresponding author on reasonable request.

## Abbreviations

3-PGA: 3-phosphoglycerate phosphatase
AS: Asparagine synthetase
F-6-P: Fructose-6-phosphate
Fd-GOGAT: Ferredoxin-dependent glutamate synthase
G-6-P: Glucose-6-phosphate
GABA: γ-aminobutyric acid
GS: Glutamine synthetase
PEP: Phosphoenolpyruvate
