# Supplementary material for: Physiological and metabolomics analyses of young and old leaves from wild and cultivated soybean seedlings under low-nitrogen conditions
Source: BMC Plant Biol. 2019 Sep 6;19:389. doi: 10.1186/s12870-019-2005-6 (PMC6731624; doi:10.1186/s12870-019-2005-6)
Supplement: Supplementary file 1 — The growth performances in young and old leaves of two soybean varieties under LN stress. C, cultivated soybean; W, wild soybean; CK, control treatment; LN, low nitrogen stress; Up FW, aboveground fresh weight; Up DW, aboveground dry weight; Under FW, underground fresh weight; Under DW, underground dry weight. * and ** indicate significant (P < 0.05) and highly significant (P < 0.01) differences, respectively. (DOCX 13 kb) [file 12870_2019_2005_MOESM1_ESM.docx]

**Addition file 1**

**The growth performances in young and old leaves of two soybean varieties under LN stress.**

| Parameters | C | | W | | Fold changes Log_2_^(LN/CK)^ | |
| --- | --- | --- | --- | --- | --- | --- |
|  | CK | LN | CK | LN | C | W |
| Shoot height（cm） | 60.00 | 40.00 | 102.00 | 96.00 | -0.58^**^ | -0.09^*^ |
| Root length（cm） | 34.50 | 24.00 | 29.50 | 36.50 | -0.52^*^ | 0.31^*^ |
| Up FW（g） | 65.25 | 45.60 | 55.60 | 45.90 | -0.52^**^ | -0.28^*^ |
| Up DW（g） | 13.53 | 8.11 | 11.98 | 8.48 | -0.74^*^ | -0.50^*^ |
| Under FW（g） | 21.40 | 14.00 | 18.10 | 12.45 | -0.61^*^ | -0.54^*^ |
| Under DW（g） | 5.43 | 2.09 | 6.37 | 3.57 | -1.38^*^ | -0.84^*^ |

C, cultivated soybean; W, wild soybean; CK, control treatment; LN, low nitrogen stress; Up FW, aboveground fresh weight; Up DW, aboveground dry weight; Under FW, underground fresh weight; Under DW, underground dry weight. * and ** indicate significant (P < 0.05) and highly significant (P < 0.01) differences, respectively.
