# Supplementary material for: Physiological and metabolomics analyses of young and old leaves from wild and cultivated soybean seedlings under low-nitrogen conditions
Source: BMC Plant Biol. 2019 Sep 6;19:389. doi: 10.1186/s12870-019-2005-6 (PMC6731624; doi:10.1186/s12870-019-2005-6)
Supplement: Supplementary file 3 — Total ion current chromatograms of two genotypes soybean seedling leaves extracts obtained from GC-MS. A: W-YL-CK; B: W-YL-LN; C: W-OL-CK; D: W-OL-LN; E: C-YL-CK; F: C-YL-LN; G: C-OL-CK; H: W-OL-LN. (DOCX 1139 kb) [file 12870_2019_2005_MOESM3_ESM.docx]

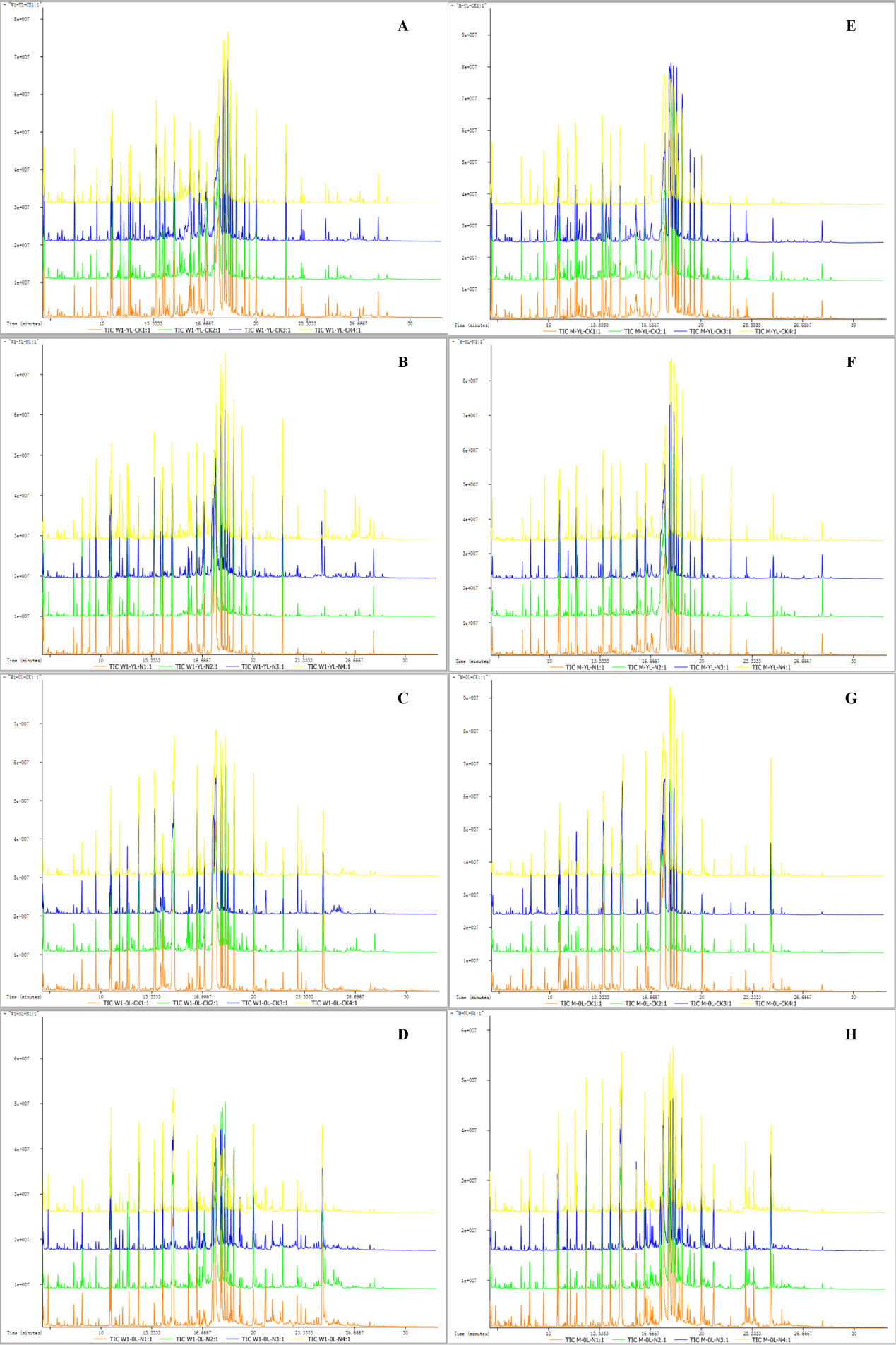


**Additional file 3 Total ion current chromatograms of two genotypes soybean seedling leaves extracts obtained from GC-MS.**

A: W-YL-CK; B: W-YL-LN; C: W-OL-CK; D: W-OL-LN; E: C-YL-CK; F: C-YL-LN; G: C-OL-CK; H: W-OL-LN.
